# Mutant polycystin-2 induces proliferation in primary rat tubular epithelial cells in a STAT-1/p21-independent fashion accompanied instead by alterations in expression of p57^KIP2 ^and Cdk2

**DOI:** 10.1186/1471-2369-9-10

**Published:** 2008-08-25

**Authors:** Kyriacos N Felekkis, Panayiota Koupepidou, Evdokia Kastanos, Ralph Witzgall, Chang-Xi Bai, Li Li, Leonidas Tsiokas, Norbert Gretz, Constantinos Deltas

**Affiliations:** 1 Department of Biological Sciences, University of Cyprus, Cyprus; 2 Institute for Molecular Anatomy, University of Regensburg, Germany; 3 Department of Cell Biology, University of Oklahoma Health Sciences Center, OK, USA; 4 Medical Research Center, University of Heidelberg, Mannheim, Germany

## Abstract

**Background:**

Autosomal Dominant Polycystic Kidney Disease (ADPKD) is characterized by the formation of multiple fluid-filled cysts that destroy the kidney architecture resulting in end-stage renal failure. Mutations in genes *PKD1 *and *PKD2 *account for nearly all cases of ADPKD. Increased cell proliferation is one of the key features of the disease. Several studies indicated that polycystin-1 regulates cellular proliferation through various signaling pathways, but little is known about the role played by polycystin-2, the product of *PKD2*. Recently, it was reported that as with polycystin-1, polycystin-2 can act as a negative regulator of cell growth by modulating the levels of the cyclin-dependent kinase inhibitor, p21 and the activity of the cyclin-dependent kinase 2, Cdk2.

**Methods:**

Here we utilized different kidney cell-lines expressing wild-type and mutant *PKD2 *as well as primary tubular epithelial cells isolated from a PKD transgenic rat to further explore the contribution of the p21/Cdk2 pathway in ADPKD proliferation.

**Results:**

Surprisingly, over-expression of wild-type *PKD2 *in renal cell lines failed to inactivate Cdk2 and consequently had no effect on cell proliferation. On the other hand, expression of mutated *PKD2 *augmented proliferation only in the primary tubular epithelial cells of a rat model but this was independent of the STAT-1/p21 pathway. On the contrary, multiple approaches revealed unequivocally that expression of the cyclin-dependent kinase inhibitor, p57^KIP2^, is downregulated, while p21 remains unchanged. This p57 reduction is accompanied by an increase in Cdk2 levels.

**Conclusion:**

Our results indicate the probable involvement of p57^KIP2 ^on epithelial cell proliferation in ADPKD implicating a new mechanism for mutant polycystin-2 induced proliferation. Most importantly, contrary to previous studies, abnormal proliferation in cells expressing mutant polycystin-2 appears to be independent of STAT-1/p21.

## Background

Autosomal Dominant Polycystic Kidney Disease is the most common hereditary renal disorder with a prevalence of at least 1:1000 and accounts for 8%–10% for all end-stage renal failure [1]. The disease is characterized by the formation of large fluid-containing renal cysts that grow over time and destroy the renal parenchyma.

It is believed that cysts originate from tubular epithelial cells that exhibit increased proliferation and reduced differentiation. This may happen after a second somatic hit occurs that inactivates the *PKD1 *or the *PKD2 *allele inherited from the healthy parent [2-4]. Microdissection of cystic kidneys revealed that cyst growth is due to an increase in cell number and not to the stretching of the cyst wall. In addition, tubular epithelial cells cultured from ADPKD cysts display augmented levels of proliferation and upregulation of proliferation-associated genes such as c-Myc, Ki-67 and PCNA [5-8]. The role of polycystin-1 (PC-1), the protein product of *PKD1*, in the proliferation of tubular epithelium has been documented. Polycystin-1 has been implicated in a variety of pathways tied to proliferation, including G-protein signaling, Wnt signaling and AP-1. [9-12]. Direct evidence about the involvement of PC-1 in cell cycle regulation was demonstrated by the observation that PC-1 overexpression activates the JAK2/STAT-1 pathway, thereby up regulating p21^waf1 ^and inducing cell cycle arrest in G0/G1 in a process requiring functional polycystin-2 (PC-2). Based on these results it was postulated that mutations in either gene could result in deregulated growth [13].

Polycystin-2 has been implicated in cell cycle regulation mainly through its calcium channel activity and its ability to activate transcription factor AP-1 [14-16]. However, there was little direct evidence linking polycystin-2 to cellular proliferation. Recently, PC-2 was directly tied to cell cycle regulation through direct interaction with Id2, a member of the helix-loop-helix (HLH) proteins that are known to regulate cell proliferation and differentiation. Overexpression of wild-type PC-2 in kidney cell lines induced cell cycle arrest at G0/G1, through upregulation of p21 and subsequent inhibition of Cdk2 kinase activity. This process was dependent on both PC-2-Id2 interaction and PC-1-dependent phosphorylation of PC-2. Although inhibition of Id2 expression corrected the hyperproliferative phenotype of mutant cells, the contribution of p21/Cdk2 pathway on the abnormal cell proliferation was not clearly addressed [17]. In an independent study, PC-2 was shown to regulate proliferation and differentiation of kidney epithelial cells and suggested that its calcium channel activity may play an important role in this process [18].

In this study, we examined the contribution of the JAK2/STAT-1/p21/Cdk2 pathway on PC-2-dependent kidney epithelial cell proliferation. We utilized cell lines HEK293 and NRK-52E expressing wild-type and mutant PC-2 as well as primary tubular epithelial cells from a *PKD2*-mutant transgenic rat [19]. Interestingly, expression of mutant PC-2 had an effect on the aforementioned pathway only in the primary epithelial cells expressing mutant *PKD2*, but this was independent of p21. On the contrary multiple approaches provided unequivocal evidence that a different cyclin-dependent kinase inhibitor, p57, is reduced in these cells. These results suggest that p57 might be the end-point of an alternative pathway that regulates PC-2-induced proliferation in ADPKD.

## Methods

### Cell culture and isolation of renal primary epithelial cells

Human embryonic kidney 293 cells, PC-2 overexpressing cells and the rat epithelial cell line NRK-52E were maintained in DMEM medium supplemented with 10% (HEK293) or 5% (NRK-52E) fetal bovine serum (FBS).

Renal primary epithelial cells were isolated from a 7.5 week-old *PKD2 *mutant trangenic rat (TGR (CMV-h*PKD2*/1–703)), abbreviated in the text as: *PKD2*(1–703) [19]. There were two transgenic rat lines created initially, 111 and 247, expressing a truncated PC-2, owing to a STOP codon at postion 704. Of the two models 247 was chosen for further work owing to a more severe phenotype. Line 111 is not maintained at the moment [19]. The primary cells were isolated by a sequential filtration method as follows: Normal Spraque-Dawley rats and *PKD2*(1–703) rats were sacrificed following standard procedures; kidneys were extracted and minced under sterile conditions. The cell mixture was passed through a 180 μm metal sieve (Retsch, Germany) followed by filtration through a 40 μm nylon cell strainer (BD Biosciences). The retained cells were collected and passed through a second 100 μm cell strainer. The filtrate of this step comprises the tubular epithelial fraction of the kidney homogenate. Tubular epithelial cells were cultured on laminin-coated tissue culture plates and maintained in Endothelial Cell Growth Medium (PromoCell, Germany) supplemented with 5%FBS, ECGS, EGF, Hydrocortison, Amphotericin B and Gentamycin. Under these conditions the cells maintained their epithelial phenotype for at least 4 passages.

### Antibodies

The following antibodies were used in this study: mouse anti-HA, goat anti-p21, goat anti-Cdk2 (Santa Cruz, Biotechnology), rabbit-anti phospho STAT1 (Cell Signaling), rabbit anti-p57 (Santa Cruz). The rabbit polyclonal anti-PC2 (epitope corresponds to amino acids 679–742 of PC2) was previously described[20]

### Plasmids

HA-*PKD2 *(WT) was generated by cloning wild-type human *PKD2 *in pcDNA 3 (Invitrogen) plasmid. HA-*PKD2*/1–702 contains almost the entire of *PKD2 *(aa 1–702) and was constructed by the addition of a stop linker in the *PKD2 *sequence. Finally, HA-*PKD2*/R742X contains amino acids 1–742 of *PKD2*. Both, HA-*PKD2*/1–702 and HA-*PKD2*/R742X were cloned in pcDNA3 vector.

### Transient transfection and Western blot analysis

Plasmids were transfected into HEK293 and NRK-52E cells using Lipofectamine 2000 (Invitrogen) reagent according to the manufacture's instructions. Western blot analysis was performed as mentioned before [21]. Briefly, cells were lysed in Nonipet P-40 (NP40) buffer (0.1% NP40, 200 mM NaCl, 50 mM TrisCl (pH 7.4) and protease inhibitors). After centrifugation at 14,000 rpm for 5 minutes, the supernatants were collected. Total amount of protein was determined using the BCA kit (Pierce). Equal amount of protein was denatured by addition of equal volume of 2 × SDS loading buffer and heating for 30 min at 50°C. Proteins were separated on an SDS-PAGE gel. After transfer to a PVDF membrane, Western blots were developed by ECL following the vendor's protocol (Amersham).

### Cdk2 Kinase Assay

Cdk2 assay was performed as previously described [22]. Briefly, 250–500 μg of the total cellular protein was immunoprecipitated with 1 μg of Cdk2 antibody for 2 hours at 4°C. After extensive washing, the precipitate was subjected to the kinase assay in the presence of 50 mM HEPES, 7.5 mM MgCl_2_, 0.5 mM EDTA, 20 mM β-glycerophosphate, 1 mM NaF, 5 mM dithiothreitol, 100 μM ATP and 10 μCi of [γ-^32^P] ATP in a total volume of 30 μl. As a substrate, 2 μg of histone H1 (Calbiochem) were added to the reaction. The reaction was carried out at 30°C for 30 min. After elution, the supernatant was fractionated by SDS-PAGE, transferred onto a PVDF membrane and autoradiographed.

### Electrophysiology

The conventional whole-cell voltage-clamp configuration was used to measure transmembrane currents in single cells as described previously [20]. Briefly, patch-clamp recordings were obtained from single cells at room temperature using a Warner PC-505B amplifier (Warner Instrument Corp., Hamden, CT) and pClamp 8 software (Axon Instrument, Foster City, CA). Glass pipettes (plain, Fisher Scientific, Pittsburgh, PA) with resistances of 5–8 MΩ were prepared with a pipette puller and polisher (PP-830 and MF-830, respectively, Narishige, Tokyo, Japan). After the whole-cell configuration was achieved, cell capacitance and series resistance were compensated (~70%) before each recording period. From a holding potential of -60 mV, voltage steps were applied from -100 to 100 mV in 20 mV increments with 200 ms duration at 3 s intervals. Current traces were filtered at 1 kHz and analyzed off-line with pClamp 8. The pipette solution contained (in mM): 100 K-aspartate, 30 KCl, 0.3 Mg-ATP, 10 HEPES, 10 EGTA, and 0.03 GTP (pH 7.2). The extracellular solution contained (in mM): 135 NaCl, 5.4 KCl, 0.33 NaH_2_PO_4_, 1 MgCl_2_, 1.8 CaCl_2_, 5 HEPES, 5.5 glucose (pH 7.4) or 130 KCl, 1 MgCl_2_, 10 HEPES, 0.1 CaCl_2_, and 5 glucose (pH 7.4).

### Cell Cycle Analysis

Cells were seeded in six-well plates in triplicates. Upon attachment, cells were synchronized by serum starvation for 24 h followed by addition of 10% serum-containing medium for the HEK293 or 2% serum-containing medium for the primary cells, for 24 hours. Cells were harvested, fixed with 80% cold ethanol followed by treatment with 25 μg/ml Ribonuclease A (SIGMA) and 50 μg/ml propidium iodide (SIGMA) for 30 min at 37°C. After incubation the cells were analyzed by FACS.

### Gene Expression Profiling with Microarrays

Gene expression profiles of primary tubular epithelial cells (TECs) isolated from PKD2(1–703) rats and SD rats were compared. RNA isolation, cDNA and cRNA synthesis and hybridization to arrays of type Rae230A from Affymetrix (Santa Clara, CA, USA) were performed according to the recommendations of the manufacturer. Microarray data was analysed based on a mixed model analysis using JMP Genomics, version 3.0 (SAS Institute, Cary, NC, USA). Standard settings were used, except the following specifications[23]: log-linear mixed models [24], were fitted for values of perfect-matches, with probe and rat group considered to be constant and the array-id random. Custom CDF, [25] with Unigene based gene/transcript definitions different from the original Affymetrix probe set definitions was used to annotate the arrays. Microarray data were submitted to NCBI GEO , sample number [GSE11500].

### Quantitative RT-PCR

Total RNA (1 μg) was isolated from cultured cells using the Rneasy Mini kit (Qiagen) and was reverse transcribed with the Protoscript reverse transcription kit (New England Biolabs) using the VN(dT)_23 _primer as recommended by the manufacturer. As a standard for relative RNA quantification (Calibrator), 1 μg of all sample RNAs was pooled together and reverse transcribed as mentioned above. Quantitative RT-PCR (qRT-PCR) amplifications were performed with a LightCycler (Roche Molecular Biochemicals) using the same starting amount and LightCycler^® ^FastStart DNA MasterPLUS SYBR Green I reagents in a standard volume of 20 μl. Real-time detection of fluorimetric intensity of SYBR Green I, indicating the amount of PCR product formed, was measured at the end of each elongation phase. Fluorescence values measured in the log-linear phase of amplification were considered using the second-derivative-maximum method of the LightCycler Data Analysis software (Roche Molecular Biochemicals). Relative quantification was performed using serial dilutions of the Calibrator cDNA to provide a standard curve for each run. For all experiments, the standard curve had an error of below 5% and extended over the relative quantities of all individual samples.

Genes whose differential expression was tested by gene-specific qRT-PCR analysis were rat p57 (forward primer: TGATGAGCTGGGAGCTGAG and reverse primer: TGGCGAAGAAGTCAGAGATG) and Cdk2 (forward primer: TGTGGCGCTTAAGAAAATCC and reverse primer: CCAGCAGCTTGACGATGTTA). Differences in the quantity of starting material were compensated by normalization with the housekeeping genes HPRT (forward primer: CTCATGGACTGATTATGGACAGGAC and reverse primer: GCAGGTCAGCAAAGAACTTATAGCC) and GAPDH (forward primer: GTATTGGGCGCCTGGTCACC and reverse primer: CGCTCCTGGAAGATGGTGATGG). Normalized fold-changes between mutant and normal samples were calculated by the REST XL software.

### Data Analysis and Statistics

Data are reported as means ± SEM. Comparisons between multiple groups were performed using single-factor ANOVA, and secondary comparisons were performed using the Tukey test. Statistical analysis was performed using the SPSS statistical software package. For electrophysiology experiments, statistical analysis was employed with the SigmaStat (Chicago, IL) software. Data were reported as means ± SEM. Due to high variability in cells transfected with wild type *PKD2*, statistical significance was determined by the Mann-Whitey Rank Sum test. Differences were considered significant at p < 0.05 if not stated otherwise (patch clamp and gene expression profiling. Gene expression statistical analysis is described above.

## Results

### Generation of stable clones expressing wild-type and mutant PKD2 in HEK293 cells

**Figure 1:**
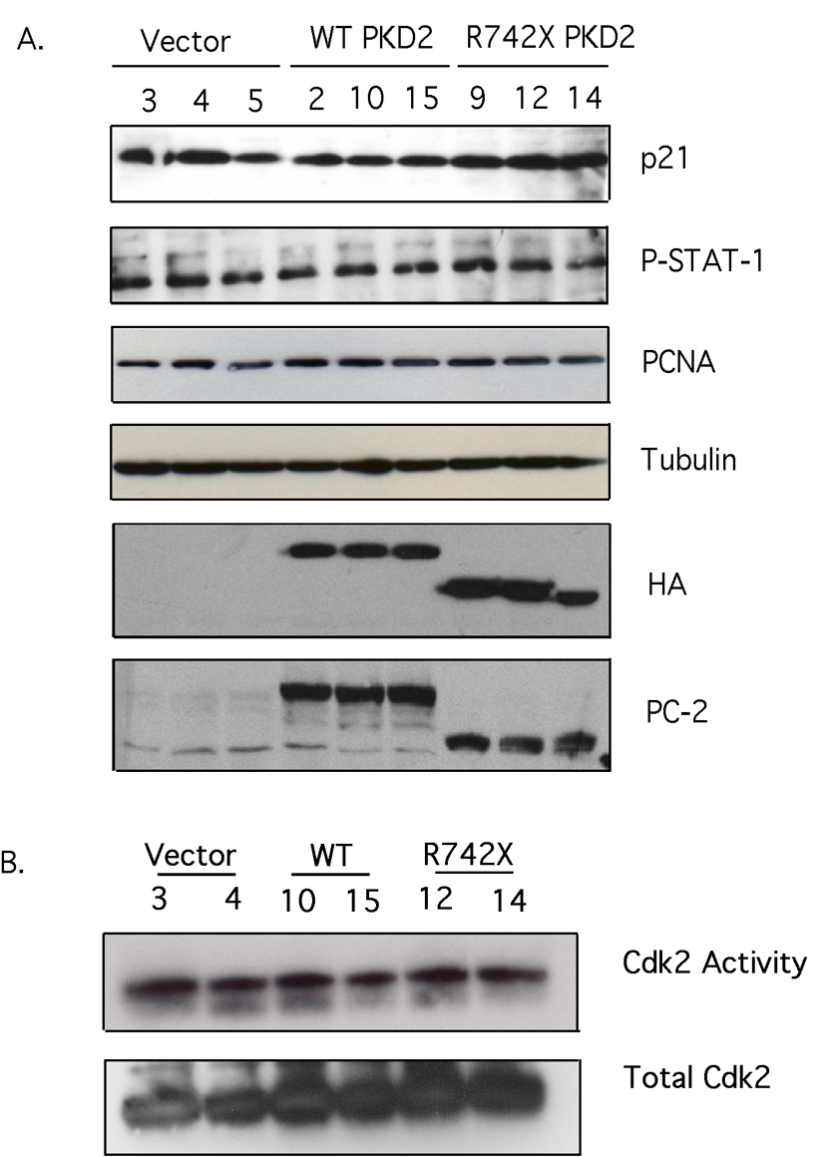
**Expression of wild-type or mutant PC-2 does not affect proliferation or STAT-1/p21/Cdk2 activity in HEK293 cells**. (A) Whole cell lysates containing equal amounts of protein from three stable individual clones of each transfectant (Vector-only, HA-WT *PKD2 *and HA-R742X *PKD2*) were analyzed by Western blotting for expression of p21, phosphorylated STAT-1, PCNA, β-tubulin, HA and PC-2. (B) Cdk2 immunoprecipitates from two clones of each transfectant were subjected into an in vitro Cdk2 kinase assay using Histone 1A as substrate. Equal amount of Cdk2 was confirmed by immunoblotting the precipitates with anti-Cdk2 antibody. Data are representative of five independent experiments.

To test the role of *PKD2 *in renal cell proliferation and specifically on the p21/Cdk2 pathway, we generated a series of HEK293 cell lines with stable expression of hemaglutinin (HA)-tagged wild-type human *PKD2 *(WT *PKD2*), HA-tagged mutant *PKD2 *(R742X *PKD2*) and a selectable marker (Vector). The R742X *PKD2 *encodes for a truncated PC-2 lacking the polycystin-1 (PC-1) interacting region at the carboxy-terminal of the protein. R742X, is a disease-causing PC-2 mutant firstly identified in a Greek-Cypriot family with Polycystic Kidney Disease type 2 [26-28]. Three individual clones were isolated from each transfectant and used for further experimentation. Immunoblotting of whole cell lysates from the selected clones with an HA antibody, showed good expression of HA-tagged WT *PKD2 *and HA-tagged R742X *PKD2 *(Figure 1A). The same lysates were immunoblotted with anti-PC-2 antibody to demonstrate that we indeed have PC-2 overexpression in these clones. As seen in figure 1A, endogenous PC-2 is barely detectable by Western blot analysis in vector-only and R742X *PKD2 *transfectants. The lower molecular weight band detected most likely represents a non-specific band detected with the anti-PC-2 antibody, since it is detected on vector-only transfectants and untransfected cells (Figure 1A and data not shown).

We used these tools to test the effect of wild-type and mutant PC-2 expression on the JAK2/STAT-1/p21/Cdk2 pathway, as it was previously implicated in its regulation by showing that overexpression of wild type *PKD1 *activates JAK2 kinase, which in turn phosphorylates STAT-1 [13]. Lysates from synchronized clones were immunoblotted with an anti-phospho-STAT-1 antibody, which detects the expression of serine phosphorylated STAT-1, and an anti-p21 to detect endogenous p21 expression. As shown in figure 1A, p21 levels and STAT-1 phosphorylation were unaffected by wild-type or mutant *PKD2 *expression. Equal loading was confirmed by re-probing the same membrane with anti-β-tubulin.

**Figure 2:**
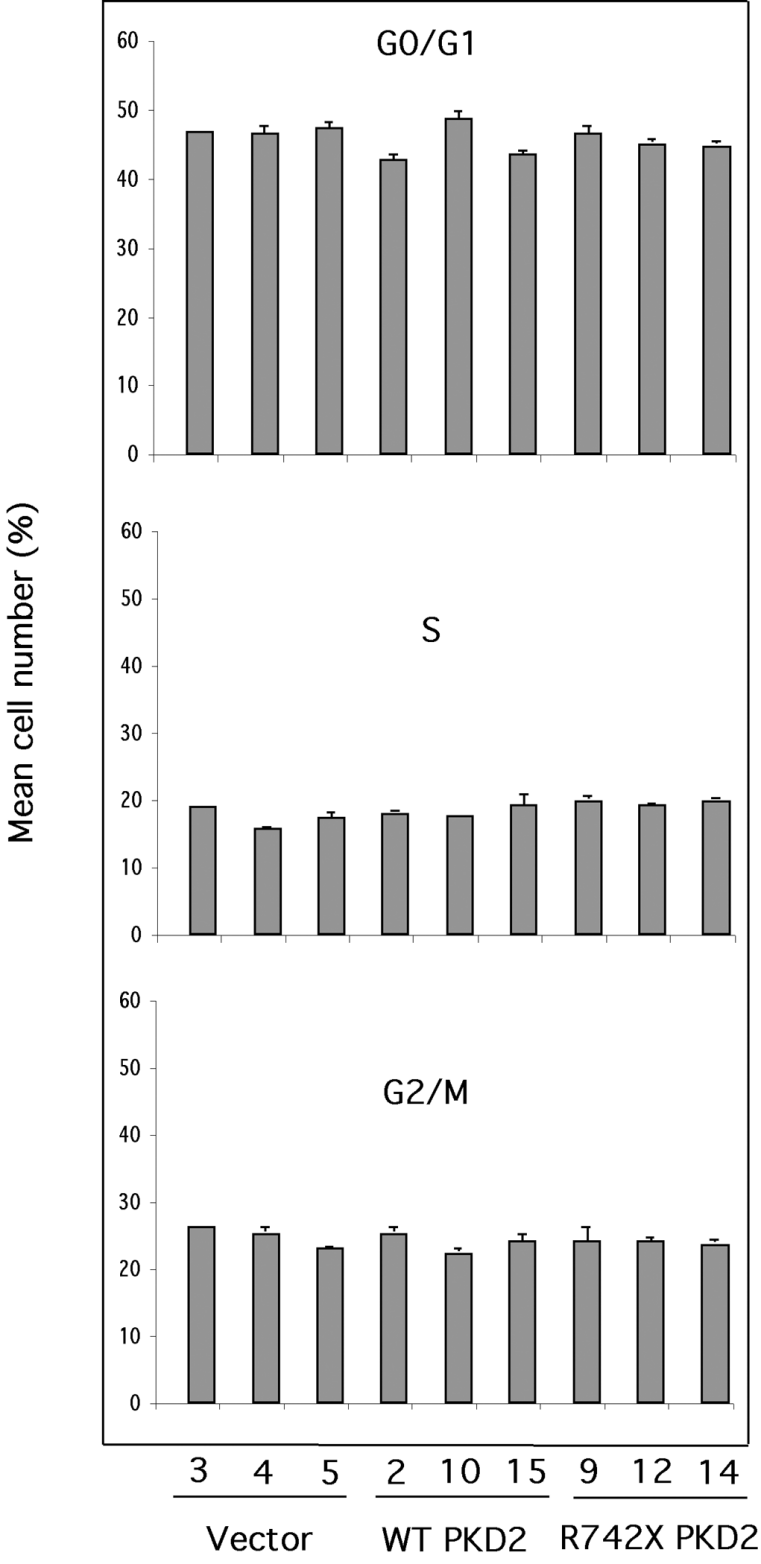
**Cell cycle profile of HEK293 clones is unaffected by expression of wild-type or mutant PC-2**. Three different clones of each transfectant were synchronized and subjected to propidium iodide cell cycle analysis by flow cytometry. The percentage of cells in each phase of the cell cycle was determined. The results are presented as mean of triplicate counts for each clone ± SEM. No statistically significant difference was detected. Analysis of serum-starved cells demonstrated that more than 90% of the live cells were arrested at G0/G1 phase confirming successful synchronization of the cells cultured (data not shown).

Similarly, endogenous Cdk2 activity was equivalent among the different clones as judged by the kinase assay performed on Cdk2 immunoprecipitates from two selected clones of each transfectant. Western blot analysis demonstrated that similar amount of Cdk2 was precipitated from each clone (Figure 1B). Cell cycle analysis performed by propidium iodide (PI) staining revealed that expression of wild-type or mutant PC-2 does not alter the cell cycle profile of these cells (Figure 2). Furthermore, proliferating cell nuclear antigen (PCNA) levels were equal among the different clones (Figure 1A). Collectively, the results suggest that expression of wild-type and mutant *PKD2 *has no effect on the proliferation of HEK293 cells.

To determine whether mislocalization of exogenous WT and R742X PC-2 is responsible for their inability to regulate cellular proliferation, we compared the sub-cellular localization of HA-tagged WT or R742X PC-2 with endogenous PC-2 by immunofluoresence. Both HA-tagged WT and R742X PC-2 were detected at the same subcellular compartments (endoplasmic reticulum and plasma membrane) as the endogenous PC-2 (data not shown).

**Figure 3:**
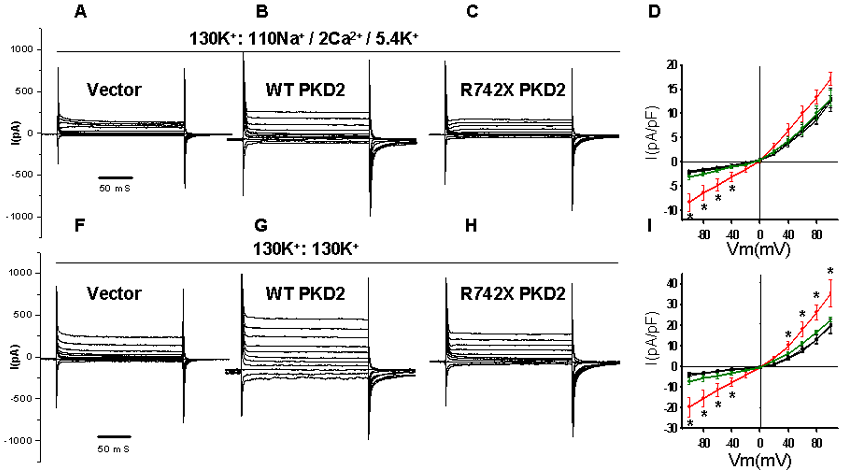
**Functional expression of *PKD2 *in HEK293 cells**. Whole cell step currents in vector-only (A and F), wild type *PKD2*- (B and G), or *PKD2*(742X)-stably transfected HEK293 cells (C and H) in normal extracellular tyrode solution (130K^+^:135Na^+^/2 Ca^2+^/5.4 K^+^) (A-C) or symmetrical K^+ ^(130K^+^:130K^+^) (F-H). Current-voltage (I–V) curves derived from a step protocol in untransfected (black squares), mock-transfected (vector control) (black circles), WT *PKD2*- (red circles), or *PKD2 *(R742X)-stably transfected HEK293 cells (green circles) in normal extracellular tyrode solution (D) or symmetrical K^+ ^(I). ''*'': p < 0.05.

ER-localized PC-2 is known to function as a Ca^2+^-activated intracellular Ca^2+ ^release channel while plasma membrane-associated PC-2 is believed to function as a nonselective cation channel [29-31]. Previous work has demonstrated that *PKD2 *overexpression augmented the amplitude of whole cell currents in renal epithelial cells [20]. To test the effectiveness of the expressed WT *PKD2 *in HEK293 cells we performed whole cell current measurements in vector-only, WT *PKD2 *and R742X *PKD2 *clones. Functional expression of transfected wild type *PKD2 *in HEK cells has been shown [32]. Figure 3 shows that stable expression of wild type *PKD2 *in HEK cells resulted in a significant increase in the current amplitude of whole cell inward currents recorded either in normal extracellular tyrode solution or symmetrical K^+ ^(Figure 3). Outward currents were larger in WT *PKD2 *expressing cells compared to untransfected, mock-transfected, or R742X *PKD2*-transfected cells in symmetrical K^+^. *PKD2*-mediated K^+ ^currents were larger compared to Na^+^/Ca^2+ ^currents as was expected for *PKD2 *which shows higher permeability to K^+ ^compared to Na^+ ^or Ca^2+ ^[16,20]. Overexpression of R742X *PKD2 *did not have a significant effect on whole cell inward or outward currents in HEK293. Collectively, the electrophysiology data show that transfection of wild type *PKD2 *resulted in functional expression in HEK293 cells. However, *PKD2 *has no effect on the STAT-1/p21/Cdk2 pathway or on the proliferation status of these cells.

### Examination of the effect of wild-type and mutant PKD2 on the JAK2/STAT-1/p21/Cdk2 pathway in NRK-52E cells

HEK293 cells were generated by transformation of human embryonic kidney cell cultures with sheared adenovirus 5 DNA [33]. The cell line has an epithelial morphology and is widely used as a kidney epithelial model. Nevertheless, there is controversy as to whether these cells are of true kidney origin, since expression studies have demonstrated that HEK293 cells have an unexpected relationship with neurons [34]. For these reasons we decided to perform the same experiments in a different cell line system more closely resembling mature kidney epithelial cells, NRK-52E.

**Figure 4:**
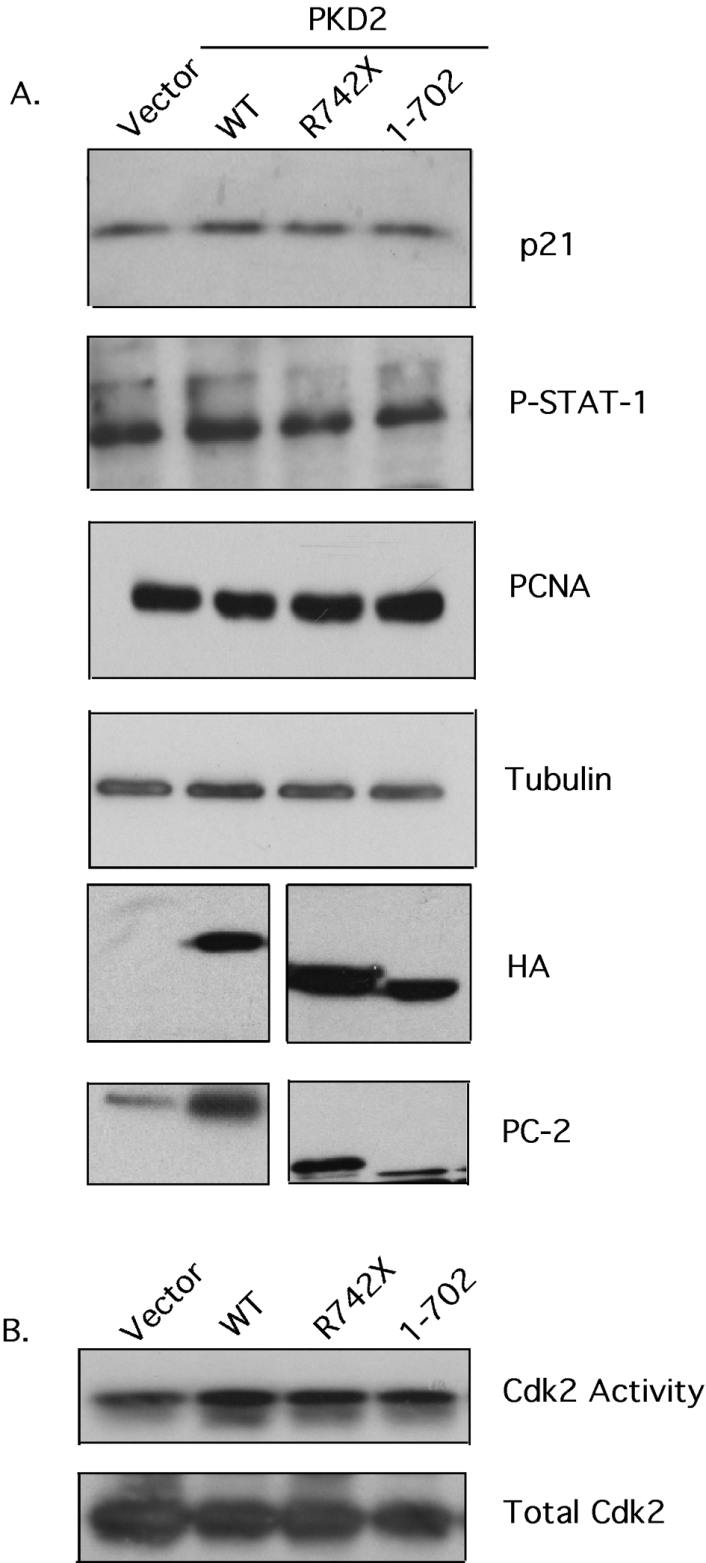
**Expression of wild-type or mutant PC-2 does not affect proliferation or STAT-1/p21/Cdk2 activity in NRK-52E cells**. **(A)**. Whole cell lysates containing equal amounts of protein from NRK-52E cells trasnsiently transfected with vector-only, HA-WT *PKD2*, HA-R742X *PKD2 *and HA-1–702 *PKD2 *were analyzed by Western blotting for expression of p21, phosphorylated STAT-1, PCNA, tubulin, HA and PC-2. A non-specific band is detected in vector-only and WT PKD2 lanes in the HA blot and co-migrates with mutated PC-2 in this cell line. (B) Cdk2 immunoprecipitates from each transfectant were subjected into an in vitro Cdk2 kinase assay using Histone 1A as substrate. Equal amount of Cdk2 was confirmed by immunoblotting the precipitates with anti-Cdk2 antibody. Data are representative of three independent experiments performed. No statistically significant difference was detected.

The rat kidney epithelial cell line, NRK-52E was transiently transfected with vector-only (CT), WT *PKD2*, R742X *PKD2 *and 1–702 *PKD2 *(a *PKD2 *mutant lacking the entire carboxy-terminal region of the protein). At 48 hours after transfection, cells were synchronized by serum starvation. Whole cell lysates were immunoblotted with anti-p21 and anti-phospho-STAT-1 antibodies. Neither p21 levels nor STAT-1 phosphorylation is affected by expression of wild-type or mutant *PKD2 *(Figure 4A). Similarly, the levels of active Cdk2 were comparable among the four transfectants. In addition to the JAK2/STAT-1/p21/Cdk2 pathway, the proliferation capacity of NRK-52E transfected with WT, R742X and 1–702 *PKD2 *appeared unaltered compared to vector only transfectants as judged by PCNA Western blot analysis. Good expression of the wild-type PC-2 and of the two truncated proteins was achieved as judged by anti-HA and anti-PC2 blotting. In summary, these results duplicate the observation in HEK293 that wild-type or mutant *PKD2 *expression do not modify the activity of the JAK2/STAT-1/p21/Cdk2 pathway.

### Renal tubular epithelial cells from PKD2(1–703) transgenic rat display augmented proliferation independent of the JAK2/STAT-1/p21/Cdk2 pathway

**Figure 5:**
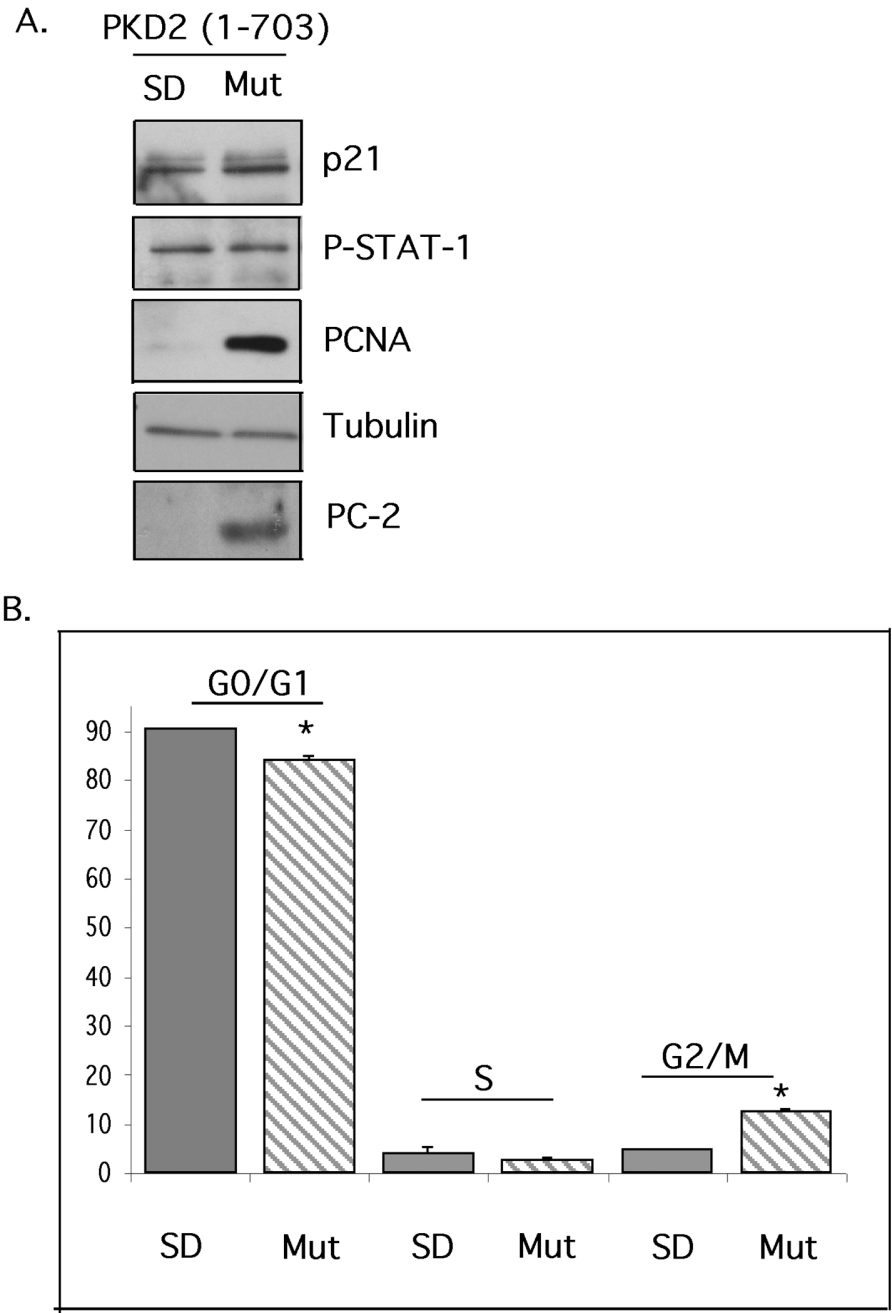
**Primary tubular epithelial cells (TECs) isolated from the kidneys of a PKD transgenic rat expressing a truncated PC-2 (*PKD2*(1–703)) display higher proliferative activity compared with TECs isolated from normal Sprague-Dawley rats**. (A). Whole cell lysates containing equal amounts of protein from TECs isolated from normal Sprague-Dawley rat (SD) and TECs isolated from PKD transgenic rat (Mut) were analyzed by Western blotting for expression of p21, phosphorylated STAT-1, PCNA, tubulin, PC-2, cadherin, vimentin and megalin. All blots are representative of experiments performed on at least two different transgenic animals. Endogenous PC-2 can be seen on long exposures that also bring out high background (not shown) (B). Cell cycle profile of normal (SD) or mutants (Mut) TECs. The results are presented as mean of triplicate counts (three independent cultures) for each animal. ± SEM (ANOVA p < 0.01. * = significant difference). The data are representative of two experiments performed using two different pairs of animals.

The unexpected but significant results above, prompted us to utilize primary renal epithelial cells obtained from a 7.5 week-old mutant *PKD2 *transgenic rat (1–703) [abbreviated *PKD2*(1–703)], expressing a truncated form of PC-2 lacking the C-terminal region of the protein. The transgenic animals manifest a cystic phenotype characterized by the formation of multiple cysts in the kidneys [19]. Tubular renal epithelial cells were isolated by sequential filtration of renal cells and cultured in low serum-containing medium. The epithelial character of the isolated cells and the absence of contaminating fibroblasts were confirmed by cadherin and vimentin expression respectively (Figure 5A).

In contrast to the cell lines examined, primary tubular epithelial cells (TECs) isolated from *PKD2*(1–703) transgenic rat, demonstrated an increase in cellular proliferation compared with their normal counterparts. Specifically, Western blot analysis on whole cell lysates demonstrated that TECs isolated from the *PKD2*(1–703) rat have significantly higher levels of PCNA than TECs isolated from normal Sprague-Dawley rats (Figure 5A). In addition, the percentage of cells in the G0/G1 phase of the cell cycle was lower in the mutant cells than in normal cells as judged by cell cycle analysis (90.6 ± 0.93 to 84.1 ± 1.28). In concert, the percentage of G2/M-phase mutant cells was higher than G2/M-phase normal cells (5.06 ± 0.31 to 12.9 ± 1.37) (Figure 5B). Despite the higher proliferative activity of mutant cells, p21 levels and STAT-1 phosphorylation remain unaltered (Figure 5A), suggesting that *PKD2*-induced proliferation is STAT-1/p21-independent.

**Figure 6:**
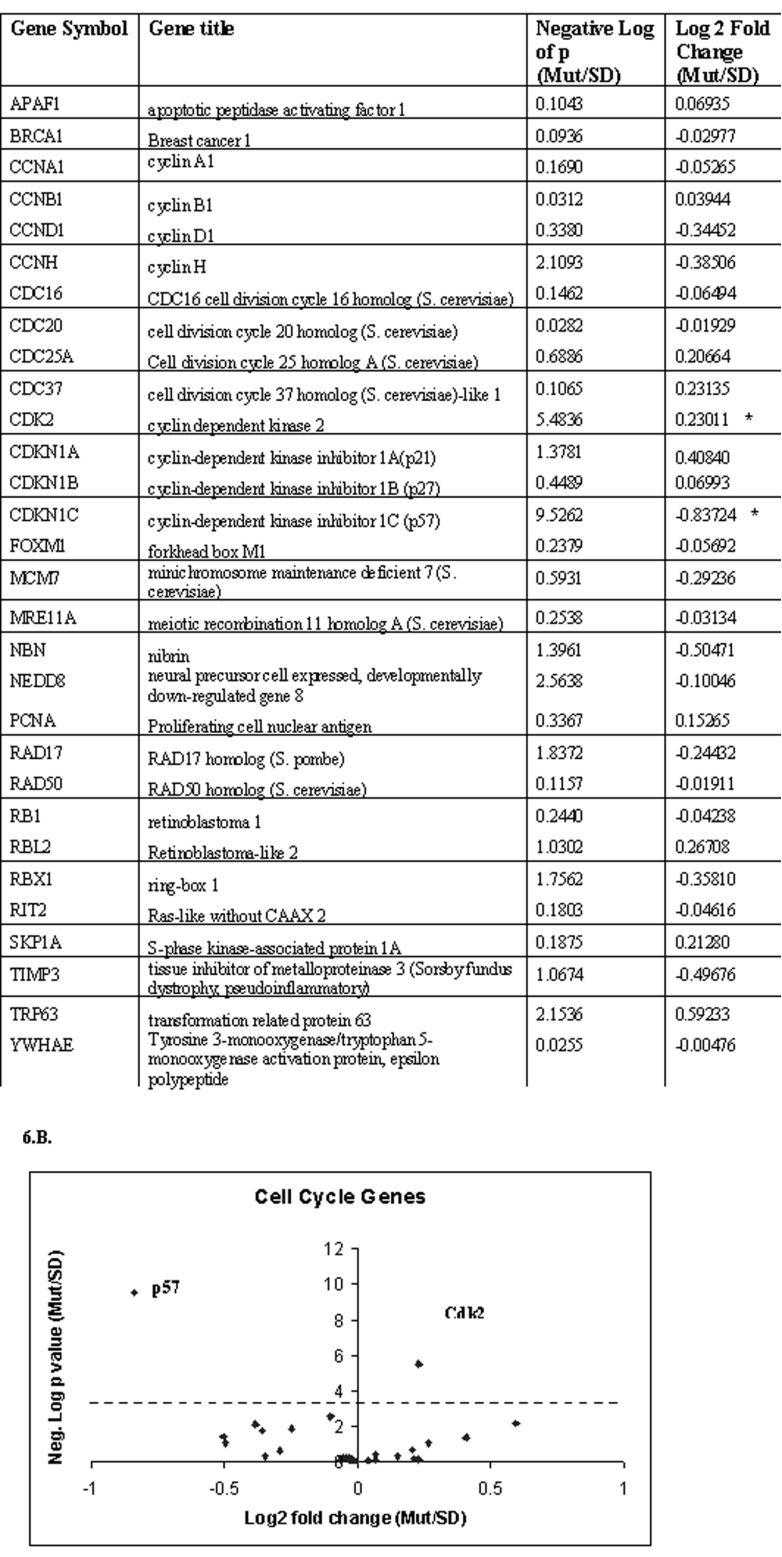
**Genome-wide expression analysis reveals differential expression of Cdk2 and p57 in TECs isolated from transgenic rat (*PKD2*(1–703))**. (A). List of cell cycle-related genes examined by microarray analysis. The (*) denotes statistical significance after Bonferroni correction. (B). Each data point on the volcano plot stands for one gene. The cutoff of p-value after Bonferroni correction is shown by the red line. Only the two significantly differentially expressed genes are labeled with their gene symbol.

We then hypothesized that alternative pathways might be responsible for *PKD2*-induced proliferation in this system. To this end, we performed a genome-wide gene expression analysis on TECs isolated from two normal Sprague Dawley rats and three *PKD2 *(1–703) rats. Differentially expressed genes were identified with ANOVA. We concentrated only on genes involved in the cell cycle regulation (Figure 6A). From all the cell cycle genes listed in figure 6A, only two differ significantly in expression between normal and mutant cells, those being Cdk2 and cyclin-dependent kinase inhibitor 1C or p57^KIP2^. On the contrary, p21 did not show any significant difference, confirming the Western blot results (Figure 6A and 5A).

**Figure 7:**
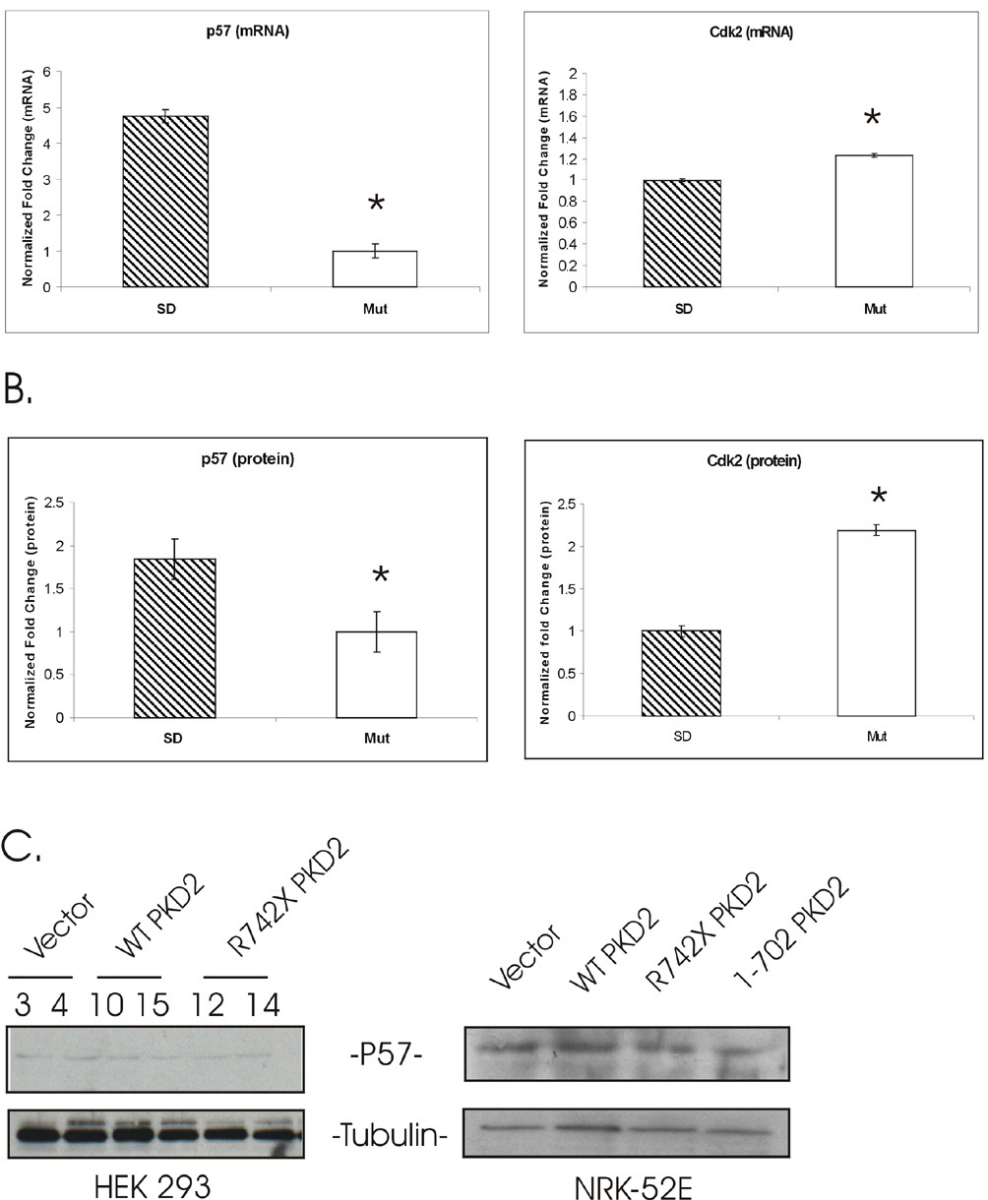
**Tubular epithelial cells (TECs) isolated from the *PKD2*(1–703) rat (Mut) have reduced p57 and augmented Cdk2 mRNA and protein compared with TECs isolated from normal rat (SD)**. (A). Real-time PCR of p57 and Cdk2 in isolated TECs. Data represent the mean of normalized fold change from three independent samples ± SEM (ANOVA p < 0.01. * = significant difference). Data were normalized using two housekeeping genes, HPRT and GAPDH. (B). Protein levels of p57 and Cdk2 represented as the mean of normalized fold change of two independent Western blot experiments ± SEM (ANOVA p < 0.05. * = significant difference). Data were normalized by β-tubulin expression. (C). p57 protein levels were determined by western blot analysis in HEK293 stable clones and in NRK-52E transfectants. As expected, protein level of p57 is not perceptibly altered.

The chip data were verified by quantitative real-time PCR analysis after normalization using two housekeeping genes, HPRT and GAPDH. In agreement with the chip data, p57 mRNA levels were downregulated in the mutant animals as compared with their normal counterparts (normalized fold change 4.7 ± 0.19). Similarly, Cdk2 mRNA levels were augmented in the mutant cells (normalized fold change 1.2 ± 0.015) (Figure 7A). Cdk2 protein upregulation and p57 protein downregulation were also verified by immunoblotting. Consistent with the microarray data, Cdk2 protein levels were significantly elevated in mutant primary cultures (normalized fold change 2.2 ± 0.06). Similarly, p57 levels were downregulated in mutant TECs (normalized fold change 1.9 ± 0.2) (Figure 7B). On the contrary, Western blot analysis demonstrated, as expected, that p57 protein levels remain unchanged in HEK293 stable clones and NRK-52E transfectants (Figure 7C). It should be noted that p57 levels in the cell lines examined is expressed at very low levels and it was barely detectable by Western blot. Given that in the *PKD2*(1–703) transgenic rat the cysts originate predominantly from the proximal tubule segment of the nephron, we wanted to exclude the possibility that proximal tubule cells are overrepresented in the primary mutant TECs culture, thus confounding the interpretation of the results. In order to do that, lysates from normal and mutant TECs were immunobloted with anti-Megalin antibody, a proximal tubule marker [35]. As shown on figure 5A, Megalin protein levels were equivalent among normal and mutant TECs suggesting that the proportion of cells of proximal origin was comparable among the different cultures and did not create a sampling bias.

## Discussion

Various studies on renal cystic tissues and cell lines demonstrated that altered regulation of tubular epithelial cell proliferation is a key factor in the pathogenesis of ADPKD. What remains unclear is the timing of the misregulated growth as well as the pathways involved. Recently, in an attempt to answer these questions a number of groups provided evidence for the involvement of Cdk2 in the process of cystogenesis. Progression through the cell cycle is regulated by a family of cyclin-dependent kinases (CKs) whose activities are controlled by the relative ratio of cyclins and Cdk inhibitors (CKIs) [36,37]. There are two classes of CKIs in mammals, the p21^CIP1 ^and p16^INK4 ^families. Members of the p16^NK4 ^family bind and inhibit only Cdk4 and Cdk6 kinases [38]. In contrast, members of the p21^CIP1 ^family (p21^CIP1^, p27^KIP1 ^and p57^KIP2^) inhibit all G1/S phase CDKs. The transition of cells from the G0/G1 into the S phase of the cell cycle involves the activities of Cdk2, Cdk4 and Cdk6 [37].

Bhunia et. al. were the first to address the role of CDKs in PKD-induced proliferation. Specifically, they demonstrated that one of the functions of the polycystin-1/2 complex is to regulate the JAK/STAT pathway and consequently control cellular proliferation. They showed that overexpression of wild-type polycystin-1 can activate JAK2/STAT-1, a process that resulted in upregulation of the CKI p21^waf1^. As expected, increase in p21 levels led to inhibition of Cdk2 and cell cycle arrest. The ability of polycystin-1 to modulate Cdk2 activity was dependent on polycystin-2. These results implied that compromised polycystin-1 activity is expected to have the opposite effect, thus explaining the abnormal proliferation observed in ADPKD cystic cells.

An independent study addressed directly the role of PC2 in cell cycle regulation and Cdk2 activity. It was demonstrated that PC2 could directly interact with Id2, a member of the HLH family that is known to control cell proliferation and differentiation. The direct association of PC2 with Id2 was shown to regulate the nuclear translocation of Id2 and thus modulate the cell cycle through the Id2/p21/Cdk2 pathway [17]. Based on these results a model was proposed according to which PC1 can increase PC2 phosphorylation leading to enhanced Id2/PC2 interaction and reduced Id2 nuclear import. This in turn, prevents Id2 repression of E-box-dependent activation of transcription of genes such as p21. Increased p21 will inhibit Cdk2 activity and arrest the cells at G0/G1 phase of the cell cycle. At the same time PC1 can lead to Cdk2 inhibition independent of Id2 through the JAK/STAT pathway. Based on this model mutations in either PC1 or PC2 can disrupt these pathways leading to abnormal cell proliferation [17]. A recent report also demonstrated reduced levels of p21 in human and animal PKD tissues as well as in affected cell lines implying a role of p21/Cdk2 in cystogenesis [39].

In this study we attempted to examine further this hypothesis. We generated stable clones expressing either wild-type or mutant R742X *PKD2 *in HEK293. To our surprise, overexpression of wild-type PC2 did not affect proliferation of these cells. Cell cycle profile analysis, PCNA, p21 expression levels and Cdk2 activity remained unchanged among different transfectants. The reason for this discrepancy remains unclear given that the same cell line and similar experimental conditions were used in the previous studies [13,17]. In order to eliminate the possibility that the exogenously expressed wild-type *PKD2 *was not functional, we performed whole cell current measurements in vector-only, WT *PKD2 *and R742X *PKD2 *clones. As expected, HEK293 clones expressing wild-type *PKD2 *displayed an increase in the current amplitude of whole cell inward and outward currents recorded either in normal extracellular tyrode solution or symmetrical K^+^. Such result excludes the possibility that an inactive PC-2 was expressed in HEK293 cells. In addition, absence of phenotype could not be attributed to the mislocalization of the expressed protein as determined by immunofluorescent analysis.

In an attempt to clarify these contradictory results we utilized a different cell line system. The NRK-52E cells are "normal" rat tubular epithelial cells, thus we hypothesized that this is a more appropriate system to study PC-2-induced proliferation and STAT-1/p21/Cdk2 activation. Nevertheless, similar results were obtained with the NRK-52E cells (Figure 4). The disparity of our results compared to previous studies is puzzling. Li et. al [17], observed cell cycle arrest and Cdk2 inhibition in HEK293T cells after expression of wild-type PC-2, and not in HEK293 cells used in our study. HEK293T cell line is a derivative of HEK293 that stably expresses the large T-antigen of SV40. In these cells transfected plasmids that contain the SV40 origin are replicated to a copy number of 400–1000 plasmids/cell and therefore express the transgene at higher levels. However, this is unlikely to be the reason for the discrepancy given that high expression of wild-type and mutated PC-2 was achieved in our HEK293 clones (Figure 1) and in NRK-52E cells (Figure 4) after transient transfection.

One of the unwanted side effects of cellular immortalization might be the alteration of basal proliferation rate in cells. This can be highly significant in proliferation studies. As a result we decided to switch to a primary cell culture system. We examined the ability of mutated PC-2 (1–703) to activate the STAT-1/p21/Cdk2 pathway in primary renal epithelial cells isolated from *PKD2*(1–703) transgenic rat [19].

Isolation of TECs from the transgenic animals was performed using a sequential filtration method. Using this method we avoided any potential activation of surface receptors taking place during antibody-based isolation techniques. Purified tubular epithelial cells were cultured in low serum medium and on laminin-coated plates to avoid differentiation. The epithelial character of the cells was regularly evaluated by measuring epithelial (cadherin) and fibroblastic (vimentin) markers. TECs isolated from different animals showed augmented PCNA levels, a decrease of the G0/G1 phase cells and increase of the G2/M phase cells. This was the first time in our hands that we observed a higher proliferation activity in cells overexpressing a mutated PC-2. These results indicated that indeed PC-2 can alter cellular proliferation in renal epithelial cells, but it also suggests that such process is complicated and possibly multifactorial and can not be easily recapitulated in *in vitro *cell line systems [40]. In support of this, a recent report focused on the dynamics of cyst formation by utilizing an inducible *Pkd1 *mouse model, demonstrated that proliferation was not appreciably higher in cystic specimens than in aged matched controls. Based on their results, the authors suggested that the relationship between cellular proliferation and cyst formation may be indirect [41]. Similar data were obtained from Zebrafish studies where it was shown that increased cell number in cyctic phenotype is a secondary consequence of tubule dilation rather than the leading cause of cyst formation [42]. In our study, it appears that mutated PC-2-induced proliferation in primary cells proceeds independently of the STAT-1/p21 pathway since there is no change in the levels of p21 or on STAT-1 phosphorylation. Based on these results it is clear that in the rat system we investigated, PC-2-induced proliferation proceeds through an alternative pathway other than STAT-1/p21.

Using gene expression profiling we were able to identify a candidate that may mediate the PC2-induced proliferation in *PKD2*(1–703) rat. Among all the cell cycle related genes, only two showed misregulation in TECs isolated from diseased rats, cyclin-dependent kinase inhibitor 1C (p57kip2) and Cdk2. The p57 kip2 belongs to the p21^WAF/Cip1 ^family. Studies have shown that p57 binds tightly to the G1 and S phase kinases, cyclin E/Cdk2, cyclin D2/Cdk4, cyclin A/Cdk2 and to a lesser extent to cyclin B/Cdc2 and effectively inhibits their activity [43]. An important difference between p57 and the other members of the family, is that p57 is not regulated by p53 but by p73 [44-46].

We observed a downregulation of p57 at both mRNA and protein levels in mutant cells with the absence of any change in p21 levels. This possibly signifies that PC-2 might alter cellular proliferation through p57/Cdk2 in these cells. It is possible that expression of mutant PC-2 can result in p57 downregulation by augmenting Id2 nuclear import and subsequent inhibition of p57 transcription [17]. This hypothesis is in agreement with experiments in neural cells where it was shown that Id2 could regulate cell cycle through p57 [47].

In addition to p57 downregulation, we observed an increase in Cdk2 protein level. This is interesting since it appears that Cdk2 activity might be augmented simultaneously in two different ways (downregulation of the inhibitor and upregulation of the kinase). Whether Cdk2 increase is part of a positive feedback loop is still not known. Nevertheless, this simultaneous alteration in p57 and Cdk2 levels might result in a rapid increase in Cdk2 activity and subsequently to higher proliferation rate. A concern regarding our results might arise from the possibility that the isolated TECs are not equally representative of the various nephron segments in healthy and mutant rats, a concern however that cannot easily be addressed within the scope of this work. More specifically though, we addressed the issue of over-representation of the TECs from the proximal cysts by showing similar levels of a proximal tubule marker, megalin expression in normal and mutant TECs (Fig. 5A).

In conclusion, the level of p57 contribution in the PC-2-induced proliferation in renal epithelial cells is still unclear. Future experiments will focus on identifying the pathways leading to p57 reduction and whether this decrease is necessary for PC-2-induced proliferation in renal tubular epithelial cells. We consider it of particular significance that no matter how these experiments pan-out, our study introduces a new pathway in ADPKD, through which PC-2 might lead to Cdk2 activation and increase in cellular proliferation, which is independent of STAT-1/p21. Also, once again it should be emphasized that biological systems are unpredictably complex and may exert similar effects and end results through more than one pathway. Finally a word of caution should be expressed as regards the interpretation of experiments performed on genetically grossly modified established cells lines, which are far from representing the complexity of whole organs or organisms.

## Conclusion

We have shown that p57^KIP2^, a cyclin-dependent kinase inhibitor is downregulated and cyclin-dependent kinase 2 (Cdk2) is upregulated in primary tubular epithelial cells isolated from a *PKD2 *transgenic rat. In addition, primary cells expressing mutant *PKD2 *exhibit increased proliferation compared to their normal counterparts. On the contrary, expression of mutant *PKD2 *in two kidney cell lines failed to alter cellular proliferation and p57^KIP2 ^protein levels. Most importantly, although exogenous expression of mutant *PKD2 *ablated current activity, compared to wild-type, however in cell lines or primary TECs had no effect on the STAT-1/p21/Cdk2 pathway. In conclusion this report highlights the probable involvement of p57^KIP2 ^on epithelial cell proliferation in ADPKD implicating a new mechanism for mutant polycystin-2 induced proliferation.

## Competing interests

The authors declare that they have no competing interests.

## Authors' contributions

All authors have read and approved the manuscript. KNF and PK performed most of the experiments. KNF also helped in the conception of the experimental plan and in the writing of this manuscript. EK helped in the establishment of HEK293 stable clones. RW and NG created and maintained the transgenic PKD rat. NG provided the PKD transgenic rats. LT and C–XB provided the cDNA constructs and performed the electrophysiology experiments. NG and LL designed and performed the microarray experiments. Finally CD conceived the study, supervised the work and helped in the writing of this manuscript.

## Pre-publication history

The pre-publication history for this paper can be accessed here:


